# Real Depth-Correction in Ground Penetrating RADAR Data Analysis for Bridge Deck Evaluation

**DOI:** 10.3390/s23021027

**Published:** 2023-01-16

**Authors:** Sepehr Pashoutani, Jinying Zhu

**Affiliations:** Department of Civil and Environmental Engineering, University of Nebraska-Lincoln, 1110 S 67th St., Omaha, NE 68182, USA

**Keywords:** GPR, bridge deck, depth-correction, migration

## Abstract

When ground penetrating radar (GPR) is used for the non-destructive evaluation of concrete bridge decks, the rebar reflection amplitudes should be corrected for rebar depths to account for the geometric spreading and material attenuation of the electromagnetic wave in concrete. Most current depth-correction methods assume a constant EM wave velocity in the entire bridge deck and correct GPR amplitudes based on the two-way travel time (TWTT) instead of the actual rebar depth. In this paper, we proposed a depth-correction algorithm based on the real rebar depths. To compare different depth-correction methods, we used gprMax software to simulate GPR signals in four models with various dielectric constants and conductivity. The comparison shows that the TWTT-based depth-correction method tends to over-correct GPR amplitudes so that underestimates the deterioration level of concrete decks at certain locations. Two depth-based correction methods are proposed that use migrated amplitudes and further normalize the corrected amplitude by rebar depth (attenuation rate). These methods are then applied to GPR data collected on two bridges, and the results were validated by other NDE methods and chloride concentration test.

## 1. Introduction

Ground penetrating radar (GPR) is a widely used non-destructive testing (NDT) technique to identify steel rebar corrosion and concrete deterioration in bridge decks [1,2,3,4]. GPR transmits high-frequency electromagnetic (EM) waves into materials and receives reflections from interfaces between two materials with different permittivity (dielectric constant), such as steel rebars in concrete. The amplitude of EM wave decreases with distance in concrete, and the dissipation is associated with two main factors: (a) attenuation due to electrical properties of the material and (b) geometric spreading of an electromagnetic field [5]. Geometric spreading is the widening of EM wave beam over a growing area. Both factors are functions of distance from the source. While geometric spreading effect is independent of material properties, the attenuation rate of RADAR wave is correlated with the conductivity and dielectric constant of concrete [5,6].

Rebar corrosion is often accompanied with presence of chloride, other corrosive agents, and increasing conductivity of concrete. Consequently, the amplitude attenuation increases in deteriorated areas of the bridge deck. As a perfect electric conductor (PEC), rebars in reinforced concrete reflect the total energy of EM wave. Therefore, the bridge deck condition can be assessed based on the amplitude of the reflected signals from the top-layer rebars [7]. This method is valid only when the traveling distance of EM wave is equal for all rebars. However, cover thickness in reinforced concrete bridges may not be uniform. Some bridges are constructed with side-slope drainage system which causes non-uniform cover thickness over rebars. Rebars may also deviate from their designated position during construction. To eliminate the effect of cover thickness on attenuation of the signal, many practitioners and researchers [1,5,8,9] obtained the rebar amplitude change rate with cover thickness in sound concrete. Then the amplitudes are adjusted in proportion to the rebar depths. This process is called depth-correction of GPR signals. Since the actual cover thickness is not known in many applications, the recorded time of reflections instead of rebar depth is used for this process. A GPR antenna receives a reflected signal and records its timestamps. The two-way travel time (TWTT) of an event is the time interval for the EM wave to travel from the transmitter to the reflector and then back to the receiver. TWTT from an underground dielectric interface is a function of the object’s depth and the EM wave velocity. Based on experimental data and numerical simulation, researchers have shown that the velocity of EM wave is linked to the volumetric water content of the concrete [10] and its electrical properties. With varying moisture content across the bridge deck, the wave velocity profile may not be uniform. Thus, the recorded TWTT profile of rebar reflections may not be equivalent to the cover thickness.

In this work, we proposed a real depth-correction method for GPR data analysis. We investigated various depth-correction algorithms using numerical simulations and then validated them using GPR data collected from bridges. Four simulated GPR models, representing various concrete conditions and rebar depths, are investigated in order to understand the effect of different depth-correction approaches. Previous studies [5,11] indicate that both unmigrated and migrated rebar reflection amplitudes were used to evaluate the bridge deck condition. Therefore, the effect of migration on depth-correction is also investigated in this study. These depth-correction methodologies are applied to real bridge data, and the analysis results are then compared with the Half-Cell Potential (HCP) test data collected from the same bridge. The findings of this work suggest that migration is a key factor in identifying deterioration of reinforced concrete. Additionally, the depth-correction with TWTT may over-correct the amplitude in deteriorated regions and underestimate the area of concrete deterioration in bridge decks.

## 2. Principle of Depth Correction

For concrete of good quality in dry conditions, the permittivity is low ($\epsilon_{c}\approx$ 4–6) and the conductivity is insignificant (≈$10^{-12}$–$10^{-5}$ S/m) [12]. The intrusion of chloride ions dissolved in water leads to a high dielectric constant and conductivity which increases the attenuation of the EM wave. Because water has a high permittivity ($\epsilon_{w}\approx 81$), moisture increases the permittivity of content. Equation (1) shows the velocity of the EM wave in a medium with a relative permittivity of $\epsilon_{r}$, where *c* is the speed of light in a vacuum.

(1) $$v_{m}=\frac{c}{\sqrt{\epsilon_{r}}}$$

Therefore, the TWTT of a rebar reflection depends on the relative permittivity of concrete $\epsilon_{c}$ and the cover thickness. In wet concrete, EM wave travels slower and has a larger TWTT than in dry concrete.

Because the attenuation of GPR signals depends on the travel distance, the amplitudes of rebar reflections with different cover depths should be corrected to the same depth for the evaluation of concrete deterioration and rebar corrosion. The amplitude of a plane wave at distance *d* in a medium with absolute permeability of μ, the absolute permittivity of ϵ, and conductivity of σ follows the Equation (2) with the absorption coefficient, α, defined in Equation (3). This equation is valid for non-ferromagnetic (i.e., concrete) materials, such that $\mu =\mu_{0}$ [13,14].

(2) $$A=A_{o}e^{-\alpha d},$$

(3) $$\alpha =\omega \sqrt{\frac{\epsilon \mu }{2}\left[\sqrt{1+{\left(\frac{\sigma }{\omega \epsilon }\right)}^{2}}-1\right]}$$

where $A_{o}$ is the initial amplitude of the wave at distance d=0, and ω is the frequency. The normalized GPR amplitude by the surface amplitude is expressed as dB unit

(4) $$\mathrm{dB}=20\log(A/A_{0})=-8.686\alpha d$$

It can be seen that the normalized GPR amplitude (dB) is a linear function of distance *d* and absorption coefficient α. When plotting the GPR amplitude (dB) with the distance *d*, the slope represents the absorption α, and the intercept with y-axis represents attenuation by geometric spreading, which is an inverse function of distance *d*.

**Figure 1:**
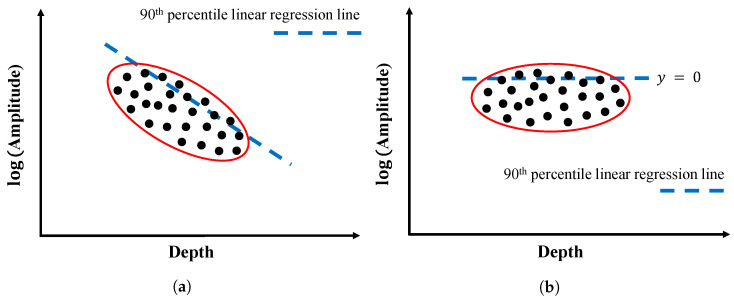
Schematic of depth correction procedure. (**a**) before correction, (**b**) after correction.

The current practice of depth-correction is to fit a 90th percentile linear regression line on the plot of GPR amplitude versus depth and obtain the attenuation rate to depth [1,8] due to geometric spreading and dielectric loss in a relatively good concrete. A schematic of this process is depicted in Figure 1a. This line represents the amplitude–depth relationship of rebars that have the top 10% of amplitudes at various depths. All data points in Figure 1a are then corrected by the 90th percentile line by subtracting the fitting line equation at different depths. Graphically, this process is equivalent to rotating the plot counterclockwise until the 90th percentile line is horizontal, as shown in Figure 1b. With this method, the amplitudes of all rebars in concrete with the same conductivity are corrected to the same depth and they can be compared for signal attenuation with the effect of depth variation.

However, this method cannot correct the attenuation caused by high conductivity. According to Equation (4), the conductivity affects the slope of the data. After correction by the 90th percentile line, the data points in high conductivity regions still have remaining slopes, i.e., the deep rebars show higher attenuation than the shallow rebars. This effect is in addition to geometric spreading and dielectric loss. To address this problem, Dinh [5] proposed to normalize conductive loss by TWTT and use this normalized conductive loss as an indication of material deterioration.

According to Equation (4), depth-correction should be based on the actual rebar depth. Because the actual depth information is not available in most cases, the TWTT is commonly used for depth correction. Strictly speaking, TWTT-based correction should be called “time-correction”, which is equivalent to the “depth-correction” only when the bridge deck has a constant wave velocity. However, velocity may vary significantly in bridge decks with deterioration, then the TWTT-based depth-correction procedure will induce error in GPR data analysis.

## 3. Numerical Models

To understand the effect of permittivity, conductivity, and depth on reflection amplitudes, four numerical models are simulated using gprMax [15], an open-source software that simulates electromagnetic wave propagation. Each model is 4 m wide with 14 PEC cylinders (rebars) embedded at varying depths from 3.8 cm to 11.8 cm with a 0.6 cm step. All side edges and bottom surface are defined as absorbing boundaries so that no reflection comes back from the edges. In each model, the combination of relative permittivity $\epsilon_{r}$ and conductivity σ represents a unique concrete condition. Table 1 shows the model parameters for each condition. These parameters are chosen within the range of reasonable values for concrete. For example, models M1 and M2 represent good quality and dry concrete, M3 for concrete in moderate condition, and Model M4 with large conductivity for deteriorated concrete.

**Figure 2:**
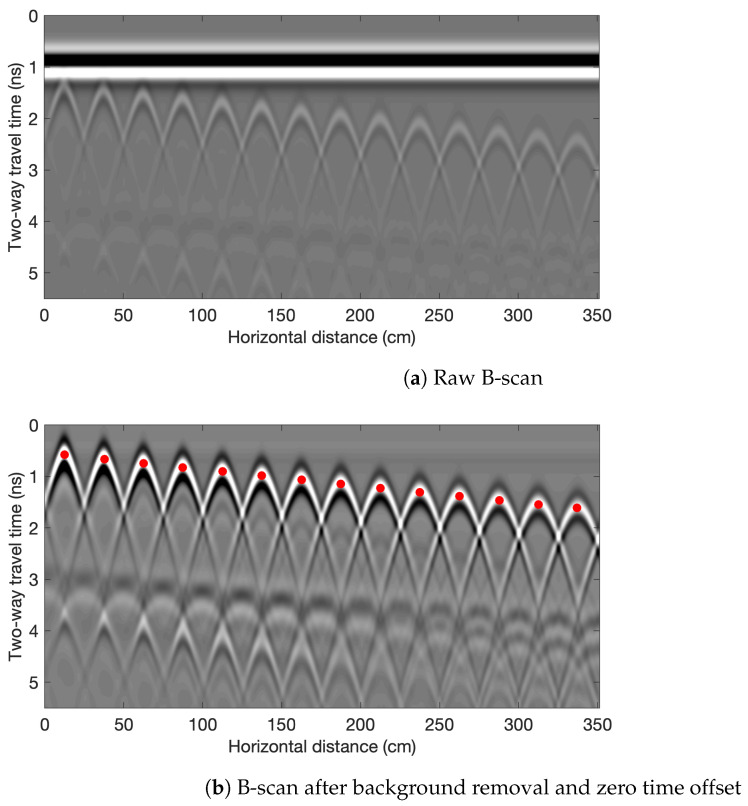

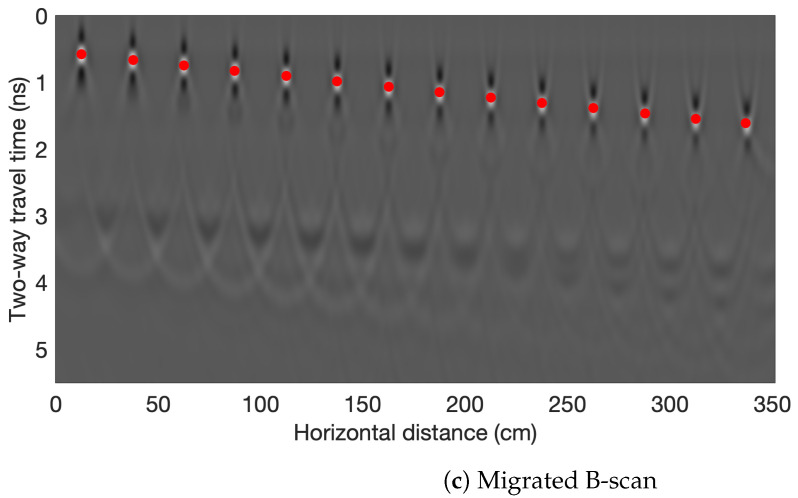
GPR B-scan pre-processing step. The shown example is for Model M1 ($\epsilon_{r}=5,\sigma =0$). The red dots mark the rebar locations.

Data processing procedure for the simulated models is illustrated in Figure 2. Before extracting the rebar amplitudes and TWTTs from the raw B-scan image in Figure 2, we need to do zero time offset and background removal. Zero time offset shifts all signals upward so that the zero time represents the time when EM wave emits from the transmitter. Background removal eliminates the strong direct wave amplitude from the B-scan and enhances the contrast of the image. Figure 2b shows the same B-scan after background removal and zero time offset. In this step, TWTTs and the amplitudes at the apex of hyperbolas are extracted. Figure 2c shows the B-scan image after migration process with selected peak amplitudes. Migration focuses the rebar reflection energy spread in the hyperbolas to a single point.

### 3.1. Depth-Correction

The following four types of depth-correction methods are presented:

- TWTT-based correction of unmigrated data (unmigrated-TWTT),
- TWTT-based correction of migrated data (migrated-TTWT),
- depth-based correction of migrated data (migrated-depth),
- depth-based correction of migrated data and then normalized by rebar depth (migrated-depth-normalized). Results of this method have a unit of dB/cm.

**Figure 3:**
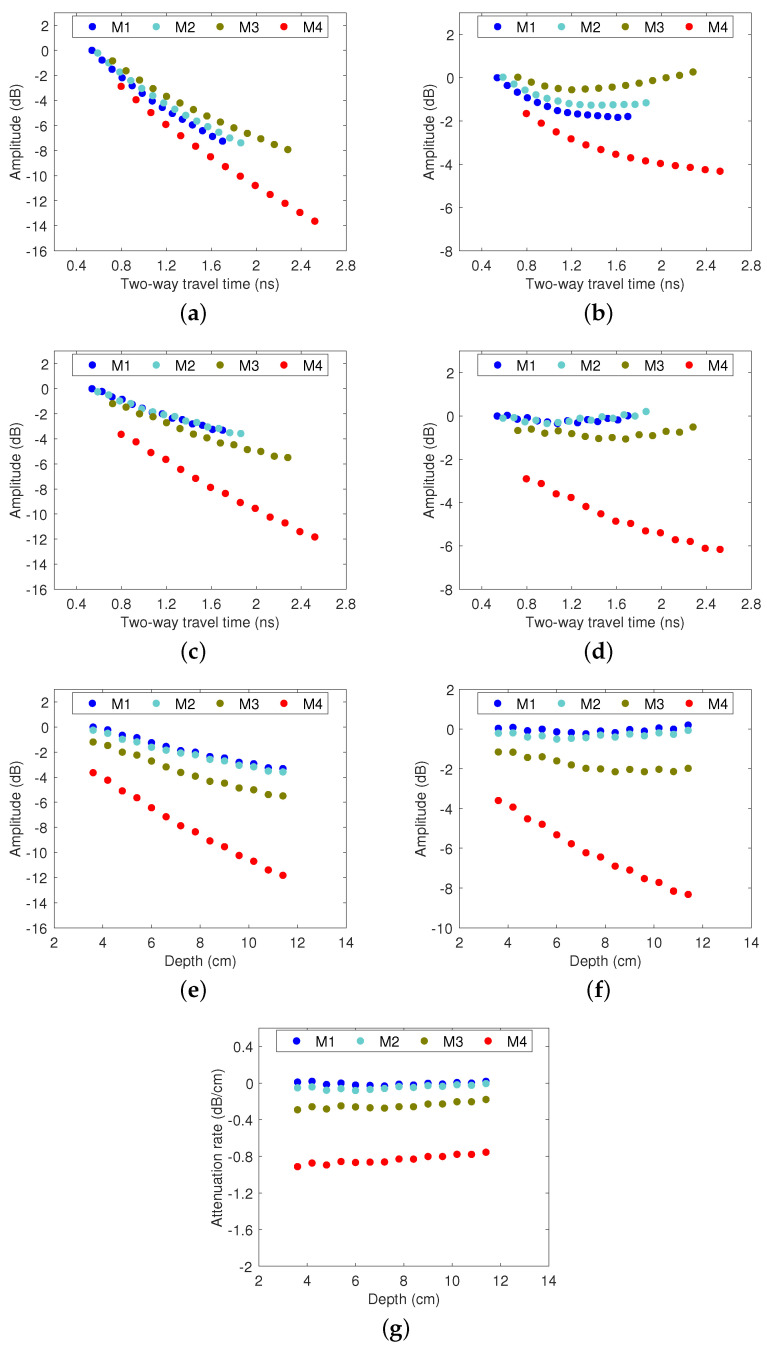
Scatter plot of shallow rebar reflection amplitudes before (**left column**) and after (**right column**) depth correction, where (**a**,**b**) unmigrated-TWTT, (**c**,**d**) migrated-TWTT, (**e**,**f**) migrated-depth, and (**g**) conductivity loss after depth correction.

Rebars in all models have the cover thickness of 3.8 cm to 11.8 cm. Figure 3 presents the GPR amplitudes for rebars at different depths. The left column Figure 3a,c,e show the uncorrected data plots for unmigrated amplitude vs. TWTT, migrated amplitude vs. TWTT and migrated amplitude vs. rebar depth. For each plot on the left column, the 90th percentile linear regression line is used to correct the amplitudes with respect to TWTT or depth. The right column Figure 3b,d,f show the corresponding corrected data plots using TWTT-correction and real depth-correction. Figure 3g gives the normalized depth-correction result, which is obtained by normalizing the data in Figure 3f by the rebar depth. An effective depth-correction process will give a very low attenuation rate for all rebars in models M1 and M2, medium level of attenuation for M3, and high attenuation for M4.

#### 3.1.1. Conventional TWTT-Based Correction

Figure 3b shows the correction results of unmigrated amplitudes with TWTT. Model M3 gives the highest amplitudes among the four cases after correction, which contradicts with the model parameters. Based on the analysis and comparison with other correction methods (Figure 3d,f,g), we conclude that using unmigrated amplitudes with TWTT correction may over-correct the amplitudes in M3 and M4 so that underestimate the areas of concrete in deteriorated condition.

#### 3.1.2. TWTT-Based Correction of Migrated Data

For depth-correction of rebars with migrated-TWTT method, EM wave velocity is required to successfully focus the energy of hyperbolas to a single point. However, in practice, the true wave velocity is unknown. In addition, the velocity profile may not be uniform in the GPR scan line due to variation in the moisture content of concrete. Most researchers make an assumption that the majority of rebars and concrete are in sound condition, and use the average wave velocity for migration analysis. In this study, we follow this procedure and use the wave velocity in models M1 and M2 for migration in all models. In Figure 3c, the migrated amplitudes are plotted against the TWTTs and the correction results are shown in Figure 3d. Comparison of these two figures shows that the rebar amplitudes in models M1 and M2 are properly corrected, and M4 has the largest attenuation. However, model M3 cannot be clearly differentiated from M1 and M2, which means that concrete in moderate condition may not be identified.

#### 3.1.3. Depth-Based Correction of Migrated Data

For depth-correction of shallow rebars with the migrated amplitude-depth method, the rebar depths must be known. In the simulation data, the EM wave velocity is calculated based on Equation (1), and then migration is performed using the true velocity for each case. In practice, the actual wave velocity over each rebar can be obtained through an automated migration process [6,16,17].

In Figure 3e, the migrated amplitudes with true velocities are plotted against the actual rebar depth. Figure 3f shows the depth-corrected amplitudes, where the amplitudes of M1 and M2 are corrected to 0 dB for all rebars, and M3 can be clearly differentiated from M1 and M2. Model M4 shows very large attenuation and the amplitude decreases with rebar depth. Therefore, the real depth-correction method can clearly distinguish areas in moderate and deteriorated concrete from sound concrete. However, the rebars in model 4 show decreasing amplitude with increasing depth, because the conductivity loss in severely deteriorated concrete cannot be fully corrected.

#### 3.1.4. Normalized Depth Correction of Migrated Data: Attenuation Rate

To correct for the depth effect on conductivity loss, we can normalize the data in Figure 3f by the rebar depth of each data point. This process gives the rate of attenuation per unit of depth with a unit of dB/cm. We can observe that all data points in Model M4 have approximately equal attenuation rates.

## 4. Implementation of Depth-Correction Methods to GPR Bridge Evaluation

The four investigated depth-correction methods are applied to GPR data collected from two bridges in Nebraska, United States, The first bridge S075 17596 has an asphalt overlay, and the second bridge S077 05693R has a concrete overlay. Multiple NDT tests were performed on these bridges. In this paper, we focus on GPR data analysis and use other NDT results as a validation of the proposed GPR data-processing method.

### 4.1. Bridge S075 17596

**Figure 4:**
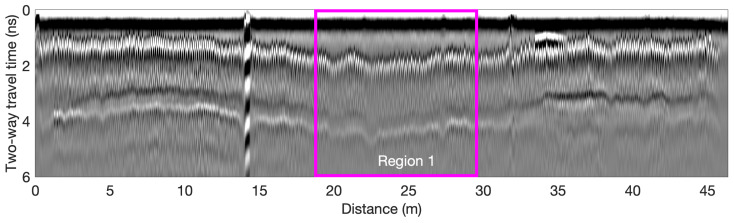
B-scan of the GPR data collected from Bridge S075 17596.

**Figure 5:**
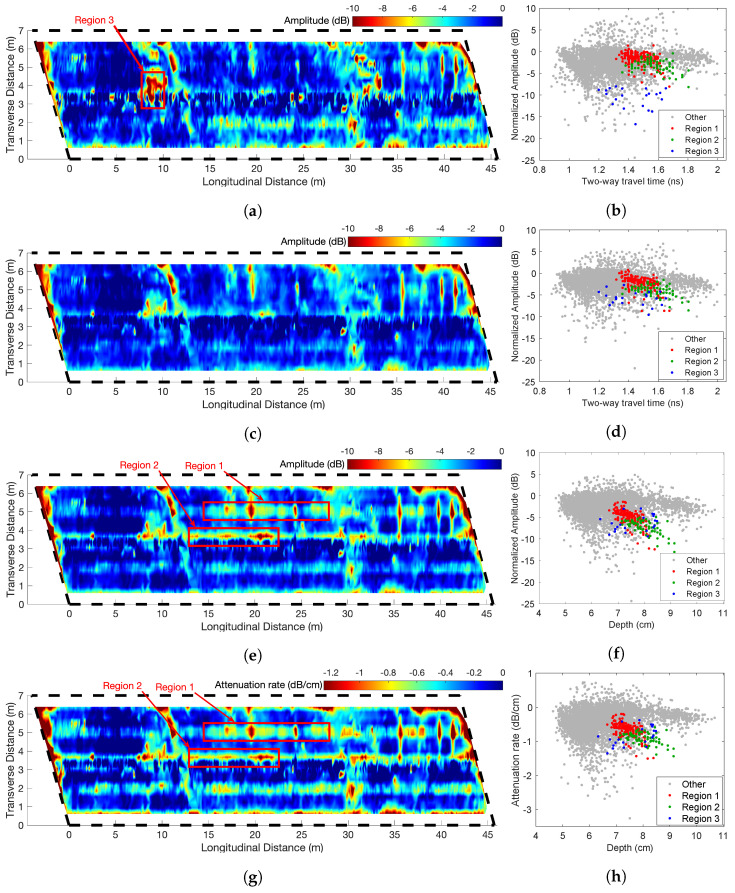
GPR amplitude map of bridge S075 17596 based on depth-correction methods using (**a**,**b**) TWTT and unmigrated amplitudes, (**c**,**d**) TWTT and migrated amplitudes, (**e**,**f**) depth and migrated amplitudes, and (**g**,**h**) attenuation rate (dB/cm) based on depth-migrated amplitudes.

The bridge S075 17596 has three spans and carries two-way traffic. The concrete bridge deck has a length of 45 m (152 ft) and a width of 12 m (40 ft), with an asphalt overlay. In 1974, the bridge was reconstructed and two shoulders were added to existing lanes. NDT tests were performed during the deck repair period. GPR and Half-Cell Potential (HCP) data were collected on bare concrete along the southbound lane and shoulder after asphalt overlay removal. Based on GPR data, the rebar spacing is 12.5 cm in the traffic lane and 25 cm in the shoulder. A GSSI SIR-4000 GPR system with a 1.5 GHz ground-coupled antenna was used to scan the bridge in the longitudinal direction. In all GPR scans, the spatial resolution was set as 3 mm in the longitudinal direction and a line spacing of 30 cm (1 ft) in the transverse direction. According to National Bridge Inventory (NBI) data, at the time of data collection, the bridge deck condition was classified as Fair [18]. Figure 4 shows a GPR B-scan collected from this bridge at the transverse distance of 5 m, which passes through Region 1 highlighted in Figure 5e. In the middle span (15–30 m), the reflections of top rebars and bottom surface have larger TWTTs and lower amplitudes than the left and right spans, which indicates lower velocity and deterioration in this range.

Figure 5 shows the result of the GPR test that was processed with four depth-correction methods: unmigrated-TWTT, migrated-TWTT, migrated-depth, and attenuation rate methods. Figure 5a,c,e,g show the corrected amplitude maps, and Figure 5b,d,f,h show the corresponding scatter plot of corrected amplitudes against TWTT or depth, respectively. The authors used the velocity analysis technique in [6] to calculate the wave velocity and estimate rebar depths. This method estimates the velocity in cover concrete for every rebar to reach the best focus of each hyperbolic rebar reflection.

Visual comparison of amplitude maps in Figure 5 shows that all four depth-correction methods in this example are able to identify major deteriorated areas. For example, all condition maps show low amplitudes occurring along the transverse joints at 0 m, 15 m, 30 m, and 45 m. Clear deterioration is observed along the horizontal lines at 3.8 m in Figure 5e,g which is also visible on Figure 5c with less clarity. These similarities show that all depth-correction methods are in general agreement in identifying severely deteriorated concrete.

**Figure 6:**
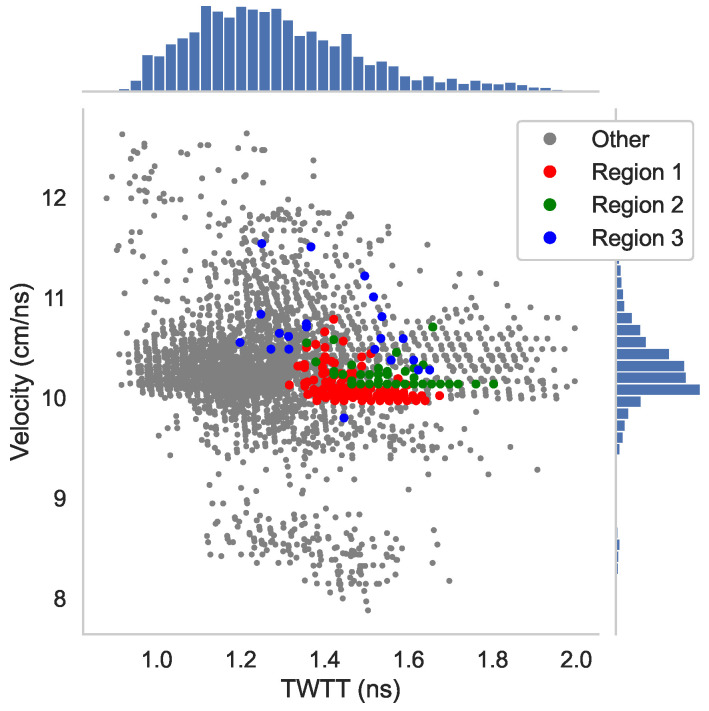
TWTT vs. velocity plot with marginal histogram (Bridge S075 17596).

On the other hand, there are several locations where these GPR maps do not give the same results. For comparison of dissimilar areas, Regions 1 and 2 are selected where the rebar reflection amplitudes are low only on Figure 5e,g. Region 3 with very low amplitudes is selected on Figure 5a. The corresponding data points in Regions 1–3 are highlighted in the scattered plots (Figure 5b,d,f,h). To understand the effect of wave velocity on the migrated amplitude, we plot TWTT versus velocity for all rebars in Figure 6, and Regions 1–3 data points are highlighted. The velocity distribution in Figure 6 indicates that the wave velocity for most data points falls within a concentrated range (9.5–11 cm/ns). The standard deviation of wave velocity throughout the entire deck is 0.566 cm/ns. The data points in all three regions have medium to high wave velocity values. Region 1 has slightly lower wave velocity than the mean value, which is consistent with observation in the B-scan image (Figure 4).

On the scattered plots, the data points in Regions 1 and 2 have low amplitude in the migrated-depth plot (Figure 5f,h), but these points have high amplitudes in Figure 5b,d, which indicates over-correction caused by TWTT-based methods. Data points in Region 3 have relatively low amplitudes in migrated amplitude plots (Figure 5d,f,h), but these points are shown with extremely low amplitudes in the unmigrated plot (Figure 5b). This discrepancy indicates the unmigrated amplitude tends to have large error for weak rebar reflections. Migration can improve the accuracy of rebar reflection amplitude. The attenuation rate map is very similar to the depth-corrected amplitude map. This is because this bridge has the majority areas in sound condition, while amplitudes in sound regions (near zero) are not affected by the depth normalization. Major findings regarding these three regions are discussed below.

- *Region 1*: The wave velocity in Region 1 is close to the average velocity of the entire bridge deck. The data points in Region 1 in Figure 5f show much lower amplitudes and a larger slope than in Figure 5b,d. This difference is related to the depth-correction method, where time-based correction methods tend to over-correct the amplitude in deterioration areas.
- *Region 2*: The cold joints between the shoulder and the driving lane are usually susceptible to chloride ingress. Time-based depth-correction results do not indicate major deterioration in the joint region. However, the migrated-depth correction method shows low amplitudes and indicates deterioration. Because the wave velocity in Region 2 is close to the average velocity, this difference is not caused by the migration process. Similar to Region 1, TWTT-based depth-correction methods over-correct the amplitude and underestimate the deterioration in the joint area.
- *Region 3*: Region 3 shows relatively low amplitudes in all maps. However, Figure 5a indicates more severe deterioration than the other amplitude maps. Because Figure 5c,e,g give similar results for Region 3, we may expect this difference is not related to the depth-correction method; instead, it is related to the migration process. For weak rebar reflection signals, the migrated amplitude is more reliable than the raw amplitude because the migration process will focus the energy spread in the hyperbola to a single point, while the weak unmigrated amplitude tends to be affected by noise.

**Figure 7:**
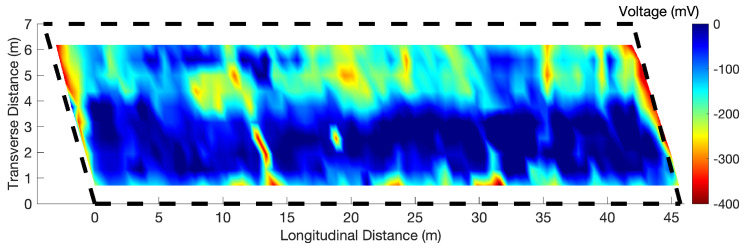
HCP voltage map of Bridge S075 17596.

After asphalt overlay removal, Half-Cell Potential (HCP) data were collected on the concrete deck surface at a 60 cm × 60 cm (2 ft × 2 ft) grid for validation. The HCP-voltage map is shown in Figure 7. Compared to the GPR maps, the HPC map missed the horizontal low amplitude line at 3.8 m in the transverse direction because of the large grid spacing used in the HCP test. The low-voltage area at 5 m in the transverse direction between 15 m to 30 m in the longitudinal direction in the HCP map matches with Region 1 in Figure 5e,g.

**Figure 8:**
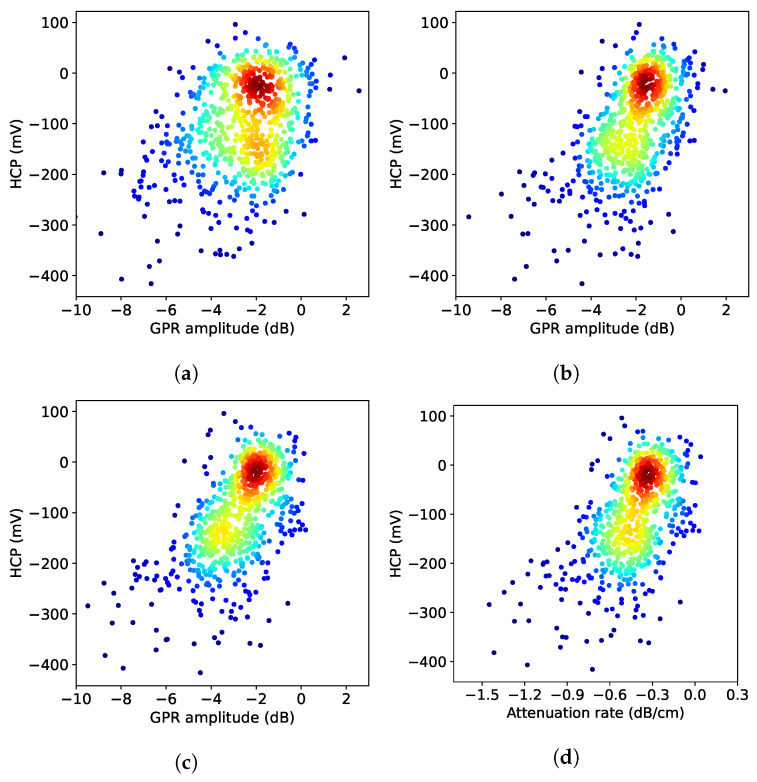
Scatter plot of HCP data and three GPR depth-corrected amplitudes on Bridge S075 17596 using (**a**) TWTT-unmigrated amplitudes (CC = 0.347), (**b**) TWTT-migrated amplitudes (CC = 0.55), (**c**) depth-corrected amplitudes (CC = 0.63), and (**d**) attenuation rate with depth-corrected amplitude (CC = 0.57). Color represents data density, and red color indicates high density.

To demonstrate a quantitative comparison between HCP and four GPR maps, we build the correlation between HCP and the three GPR depth-correction methods. Since the HCP data have a lower spatial resolution, GPR amplitudes within a 50 cm radius of each HCP grid node were extracted and averaged. The average GPR amplitudes and the corresponding HCP voltages were then plotted in Figure 8. For each depth-correction method, the correlation coefficient (CC) is calculated, which indicates the strength of the linear relationship between two sets of data. The results of this analysis indicate that all methods using migrated amplitudes give better performance than the unmigrated-TWTT method. If the actual velocity is unknown, even low-accuracy migration with a constant velocity can provide better analysis results than using the raw amplitude. The depth-corrected migrated amplitudes give the highest correlation with HCP. The correlation between the attenuation rate and HCP is slightly lower because HCP results also depend on the rebar depth.

### 4.2. Bridge S077 05693R

**Figure 9:**
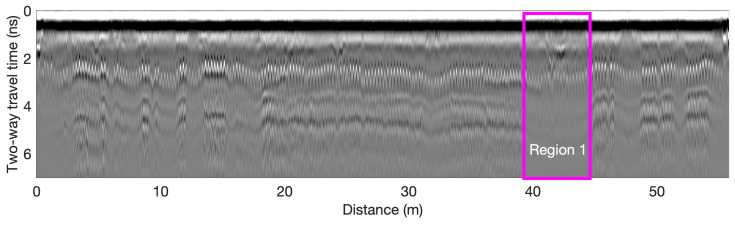
B-scan of the GPR data collected from Bridge S077 05693R.

Bridge S077 05693R is 54.9 m (180.1 ft) long with two lanes carrying one-way traffic. The bridge deck has a concrete overlay, which leads to large cover thicknesses in range of 8–14 cm. According to NBI data, at the time of data collection, the bridge deck condition was classified as Fair [18]. Based on GPR data, the rebar spacing is 12.5 cm (5 in) in the traffic lane and 25 cm (10 in) in the shoulder. A GSSI SIR-4000 GPR system with a 1.5 GHz ground-coupled antenna was used to scan the bridge in the longitudinal direction. Due to time constraint at the time of testing, only the northbound lane and the adjacent shoulder of the bridge deck was tested. In all GPR scans, the spatial resolution was set as 3 mm in the longitudinal direction and a line spacing of 60 cm (2 ft) in the transverse direction. A GPR B-scan image collected at the transverse distance of 3.3 m is shown in Figure 9. A region with obvious deterioration is highlighted.

**Figure 10:**
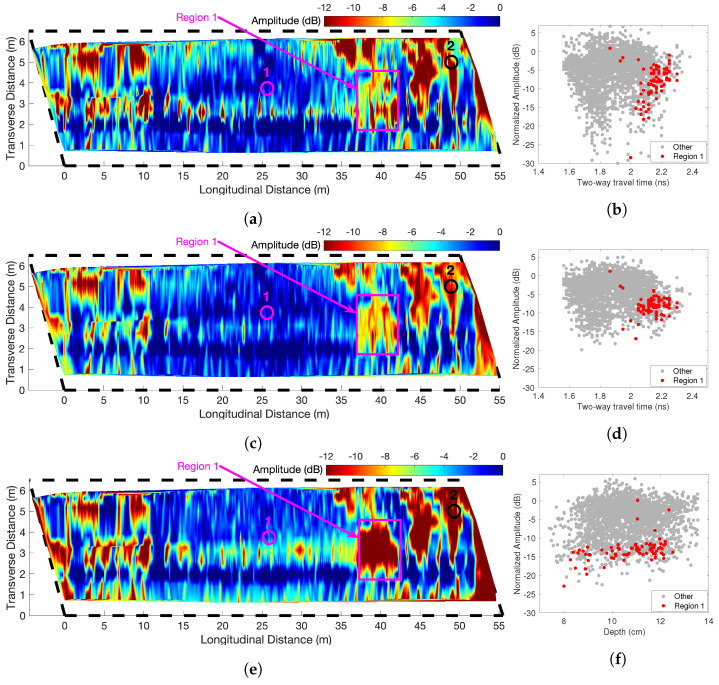

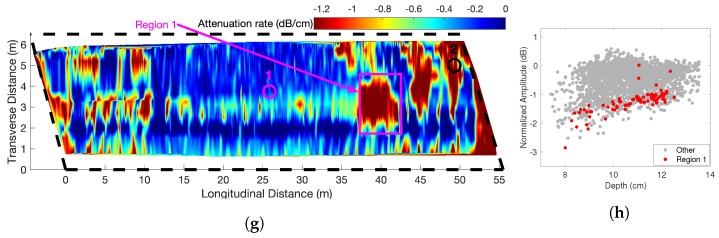
GPR condition map for Bridge S077 05693R using three depth-correction methods (**a**,**b**) TWTT and unmigrated amplitudes, (**c**,**d**) TWTT and migrated amplitudes, (**e**,**f**) depth and migrated amplitudes, and (**g**,**h**) attenuation rate (dB/cm) based on depth-migrated amplitudes. Two chloride test locations are highlighted.

Along with GPR, Vertical Electrical Impedance (VEI) [19] and high-definition imaging (HDI) [20] tests are performed. VEI is an NDE technique that uses a large-area probe which measures the impedance of the concrete cover when an alternate current is applied to the deck. Detailed data analysis for multiple NDE tests on this bridge has been reported in another paper [17]. Depth-correction of GPR amplitudes with migrated-depth method showed a good agreement between VEI and GPR [17]. In addition to VEI, at two locations on the bridge, chloride concentration testing was performed during the field testing [17]. The locations of two sampling points are shown in Table 2 and Figure 10. At these locations, concrete is pulverized at two depths of 12.5 mm and 40 mm and the powder sample is titrated in the laboratory to measure the chloride content.

GPR data were analyzed using the methods discussed in Section 3. Figure 10 shows the GPR amplitude maps generated using the four depth-correction methods. For each map, the corresponding scatter plot of corrected amplitudes against the TWTT or depth is also given. The amplitude maps are similar by visual comparison which indicates that major deteriorated areas are identifiable with all depth-corrections methods. However, the amplitude ranges within possible deterioration areas are different. Region 1 is highlighted in all maps. For example, the amplitude range inside Region 1 is [0, −18] dB in Figure 10a, while the same region shows a limited range of [−5, −13] dB in Figure 10c, [−10, −18] dB in Figure 10e. The attenuation rate range for the same region is [−2.2, −0.86] dB/cm in Figure 10g. Compared to the depth- based correction methods and VEI map [17], the TWTT-based methods over correct the amplitudes and underestimate the deterioration in this region.

A summary of chloride concentration testing is reported in Table 2. Location 1 has low chloride concentration and occurs in high GPR amplitude areas. Location 2 has a high chloride concentration and occurs at low GPR amplitude regions by all correction methods. Although the chloride concentration result confirms the validity of GPR data collection, it is unable to give further information to evaluate the depth-correction methodology.

**Figure 11:**
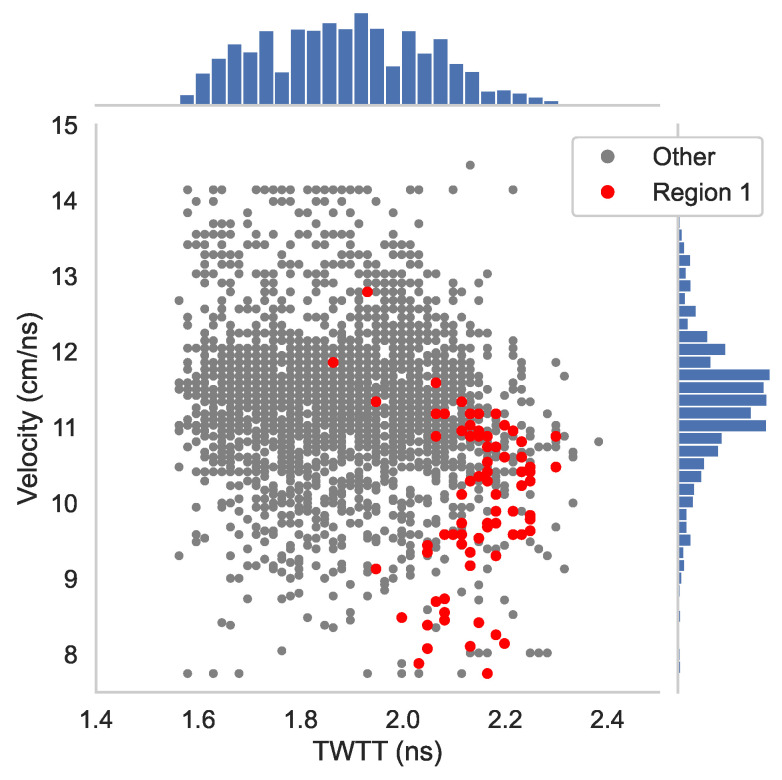
TWTT vs. velocity plot with marginal histogram (Bridge S077 05693R).

To understand the effect of wave velocity on depth-corrected amplitudes, we plotted wave velocities versus TWTTs in Figure 11. The wave velocity shows a large variation with a standard deviation of 0.977 cm/ns through the entire deck, which is larger than in Bridge S075 17596. The red dots in Figure 11 represent data points in Region 1, where the data show large TWTT in mid- to low-velocity region. As presented in the simulation results (Figure 3b,d), low velocity and high TWTT may lead to over-correction. In addition, based on velocity information, the moisture content of Region 1 concrete is high and the permittivity is in the range of 9–12. Migration with a constant velocity will cause over-migration of hyperbolas in low-velocity areas.

## 5. Conclusions

In GPR evaluation for bridge decks, the rebar amplitudes should be corrected for depth variations to obtain a reliable assessment of concrete deck conditions. Different depth-correction methods have been used in practice. Because the real rebar depths are unknown, most depth-correction methods are actually time-based corrections, i.e., the amplitudes are corrected for TWTT between the deck surface and rebars. Since deteriorated regions often have lower wave velocities than the sound areas, using TWTT for depth-correction tends to cause over-correction and under-estimation of the deterioration. In this work, we compared four depth-correction methods, unmigrated-TWTT, migrated-TWTT, migrated-depth, and attenuation rate (dB/cm), using data from four simulated models and field testing data from two bridge decks. Both shallow rebars and deep rebars are studied in the simulations and field testing. The shallow rebar models represent bare concrete decks and the deep rebar models represent bridges with overlays (asphalt or concrete). Main findings of this work are summarized as follows:

- Using real rebar depth for depth correction gives more reliable results than the TWTT-based methods. The rebar depth can be obtained from the actual wave velocity through optimized migration [6,16] and the measured TWTT. Correlation between the real depth corrected amplitudes and HCP results show good agreement on field testing data.
- Attenuation rate (dB/cm) based on depth-correction of migrated amplitude further corrects depth effect on conductivity loss, which gives the best performance in simulated results. In field testing data from two bridges, the GPR maps based on attenuation rate and depth-corrected amplitudes give similar results.
- TWTT-based depth-correction methods tend to over-correct the rebar amplitudes in deteriorated regions. Deteriorated concrete has a high permittivity and low velocity, which often causes a large TWTT. Because the depth-correction process is equivalent to the rotation of the entire dataset in the amplitude-TWTT plot, the data with large TWTT will be over-corrected in time-based depth-correction methods.
- Migration also affects the results of depth-correction. According to the simulated models and field testing data, time-based correction with unmigrated amplitudes (unmigrated-TWTT method) leads to the least accurate results. In the simulated models, this method over corrects the rebar amplitudes in deteriorated models, and in the field testing date, the corrected amplitudes show poor correlation with HCP results.
- Time-based correction using migrated amplitude still gives acceptable results. This method uses the average velocity in the sound concrete for migration processing, and plots data as migrated amplitude-TWTT format. Although this method slightly over-corrects the amplitude, the deteriorated regions can be clearly separated from the sound regions. The corrected amplitudes also show a good correlation with the HCP results. Compared to the real depth-correction method, this method needs a different threshold value for deterioration detection due to over-correction.
- Threshold values should be developed for each depth-correction method to classify deterioration. For the proposed attenuation rate method, the threshold is around 0.7 dB/cm.

## Figures and Tables

**Table 1:** Summary of gprMax model parameters.

Model	$\epsilon_{r}$	σ (S/m)	Condition
M1	5	0	Sound
M2	6	0.0002	Relatively Good
M3	9	0.002	Moderate
M4	11	0.02	Deteriorated

**Table 2:** Chloride Concentration Test Results [17].

Number	Position (X, Y) m	Depth (mm)	Chloride Conc. (%)
1	(26, 3.6)	12.5	0.09
		40	0.03
2	(49, 4.7)	12.5	0.33
		40	0.22

## Data Availability

Data available on request due to privacy restrictions.
